# Identification of genetic variants in two families with Keratoconus

**DOI:** 10.1186/s12920-023-01738-x

**Published:** 2023-11-21

**Authors:** Qinghong Lin, Xuejun Wang, Tian Han, Xingtao Zhou

**Affiliations:** 1 Department of Ophthalmology, Eye and ENT Hospital, Fudan University, No. 83 Fenyang Road, Shanghai, 200000 Xuhui District China; 2 Eye Institute and Department of Ophthalmology, Eye & ENT Hospital, Fudan University, Shanghai, 200031 China; 3 NHC Key Laboratory of Myopia (Fudan University); Key Laboratory of Myopia, Chinese Academy of Medical Sciences, Shanghai, 200031 China; 4 Shanghai Research Center of Ophthalmology and Optometry, Shanghai, 200000 China; 5 Shanghai Engineering Research Center of Laser and Autostereoscopic 3D for Vision Care (20DZ2255000), Shanghai, 200000 China; 6 Refractive Surgery Department, Bright Eye Hospital, Shanghai, 200000 China

**Keywords:** *TSC1*, *ALDH3A1*, Genetic variant, Keratoconus

## Abstract

**Background:**

This research investigated the genetic characteristic of two Chinese families with keratoconus (KC).

**Methods:**

For all people in the two families with KC, their history, clinical data, and peripheral blood were collected. One hundred healthy participants without KC and 112 sporadic KC patients were recruited as the controls. Whole exome sequencing of the genomic DNA and polymerase chain reaction were conducted for all the controls and family members to verify the variants. Functional analyses of the variants was performed using the software programs.

**Results:**

A missense tuberous sclerosis 1 (*TSC1*) variant g.135797247A > G (c.622A > G, p.Ser208Gly) was detected in family 1. A single nucleotide polymorphism (SNP) rs761232139 (p.Gly235Arg) in aldehyde dehydrogenase 3 family member A1 (*ALDH3A1*) gene was detected in family 2. The variant c.622A > G in *TSC1* and the SNP rs761232139 in *ALDH3A1* were predicted as being probably damaging.

**Conclusions:**

Novel variant c.622A > G in *TSC1* and SNP rs761232139 in *ALDH3A1* have been detected in families with KC. These two findings may play a role in the pathogenesis of KC.

**Supplementary Information:**

The online version contains supplementary material available at 10.1186/s12920-023-01738-x.

## Background

Keratoconus (KC) is a corneal disorder characterized by thinning and outward protrusion of the cornea, which results in blurred vision. The etiology of KC includes genetic, behavioral, and environmental factors [1, 2]. Among KC cases, about 5–14% have a family history of KC [3, 4]. This indicates that genetic factors are associated with the pathogenesis of KC. Autosomal dominant and recessive inheritance has been reported in KC with a family history [2, 3]. Bioinformatics studies have revealed the linkage and association of causative genes; however, few mutations have been identified for this disorder [1–4].

In this study, we used bioinformatics to investigate gene mutations in two Chinese families with KC. A novel variant in tuberous sclerosis complex 1 (*TSC1*) and a single nucleotide polymorphism (SNP) in aldehyde dehydrogenase 3 family, member A1 (*ALDH3A1*) have been detected.

## Methods

### Participants and examinations

The subjects participating in this study were 220 people including 8 family members from two Chinese KC families, 100 healthy Chinese individuals without KC, and 112 sporadic KC patients. Among the eight family members from two two-generation Chinese KC families, three were from family 1 and five were from family 2. The 100 unrelated healthy people were normal controls, who were diagnosed with refractive errors without KC and other inherited corneal disorders. The 112 sporadic KC patients (with no significant difference in gender and age compared to the normal controls,*χ*^2^ test for gender, *P* = 0.93;* t*—test for age, *P* = 0.36) were also used to verify the SNP identified in the KC members from family 2. Written informed consent of the study was obtained from all subjects. All of the participants received an ophthalmological examination, which included visual acuity testing, slit-lamp and ophthalmoscope examination, and cornea evaluation using the Scheimpflug camera system (Pentacam; Oculus Optikgeräte GmbH, Wetzlar, Germany). This investigation was reviewed and approved by the Institutional Review Board of Fudan University and was performed in compliance with the Declaration of Helsinki (approval number: 2022128).

According to the Chinese Expert Consensus on the Diagnosis and Treatment of Keratoconus (2019) [5], the clinical diagnostic criteria of KC include progressive myopia, irregular astigmatism, and poor refractive correction with glasses; corneal topography showing corneal thinning and abnormal elevation of the anterior and posterior surfaces of the cornea; and/or decreased biomechanical indexes and corneal resistance factor in a corneal biomechanical examination. The stages of KC are as follows: 1) latent stage: the contralateral eye of a confirmed KC eye, which shows typical corneal topographic features of KC and has normal vision with uncorrected visual acuity (UCVA) ≥ 1.0; 2) incipient stage: KC is confirmed with a best spectacle-CVA (BSCVA) ≥ 0.8; 3) completion stage: KC is confirmed with a BSCVA < 0.8. Typical clinical manifestations of KC are classified into three levels: Grade 1: corneal curvature (CC) < 53.0 D, corneal thickness at the thinnest point (CTTP) > 400 μm, and BSCVA < 0.8; Grade 2: CC < 55.0 D, CTTP > 300 μm, and BSCVA < 0.3; Grade 3: CC > 55.0 D, CTTP ≤ 300 μm, and BSCVA < 0.05); and 4) scar stage: scarring of the cornea after acute KC edema has subsided.

### Whole exome sequencing

Exome sequencing (ES) was performed for all participants using as previously described [6]. Briefly, genomic DNA was extracted from peripheral blood leukocytes. Then the genomic DNA from the KC subjects was used for ES and enrichment of the exonic sequences. Afterwards, data processing and analyses were conducted. Variants have been reported (e.g., transforming growth factor beta-induced) and those presented in the KC subjects at frequencies ≤ 1% were examined in the 1000 Genomes Project. Only the variants shared by family members II:1 and I:1 in family 1 or II:2, II:3 and I:2 in family 2 were taken into account as candidate mutations.

### Variant and SNP validation and cross-species conservation analysis

The variant validation and analyses were performed as previously described [6]. Briefly, the candidate mutations were confirmed using polymerase chain reaction (PCR) and Sanger sequencing in the normal controls. The PCR primers were designed using Primer3. The validation and analyses were conducted according to the NCBI VARIANT, NCBI HomoloGene, and the 1000 Genomes Project databases. Databases such as the X1000g2015aug_eas, ExAC_EAS, and gnomAD_exome_EAS were used to check the allele frequency of the variant. Scoring according to the American College of Medical Genetics and Genomics (ACMG) [7] was used for interpretation of the variant. The conserved analysis was conducted using Polyphen2 (http://genetics.bwh.harvard.edu/pph2/) and multiple sequence alignment conformational alteration was conducted using the Swissmodel (https://swissmodel.expasy.org). The SNP was checked by using the Amelie database (https://amelie.stanford.edu/) and ClinVar database (https://www.ncbi.nlm.nih.gov/clinvar/variation/2442791/). PCR and sequencing in the normal controls and sporadic patients were conducted to verify the SNP identified in the family 2.

## Results

### Clinical manifestations

**Fig. 1:**
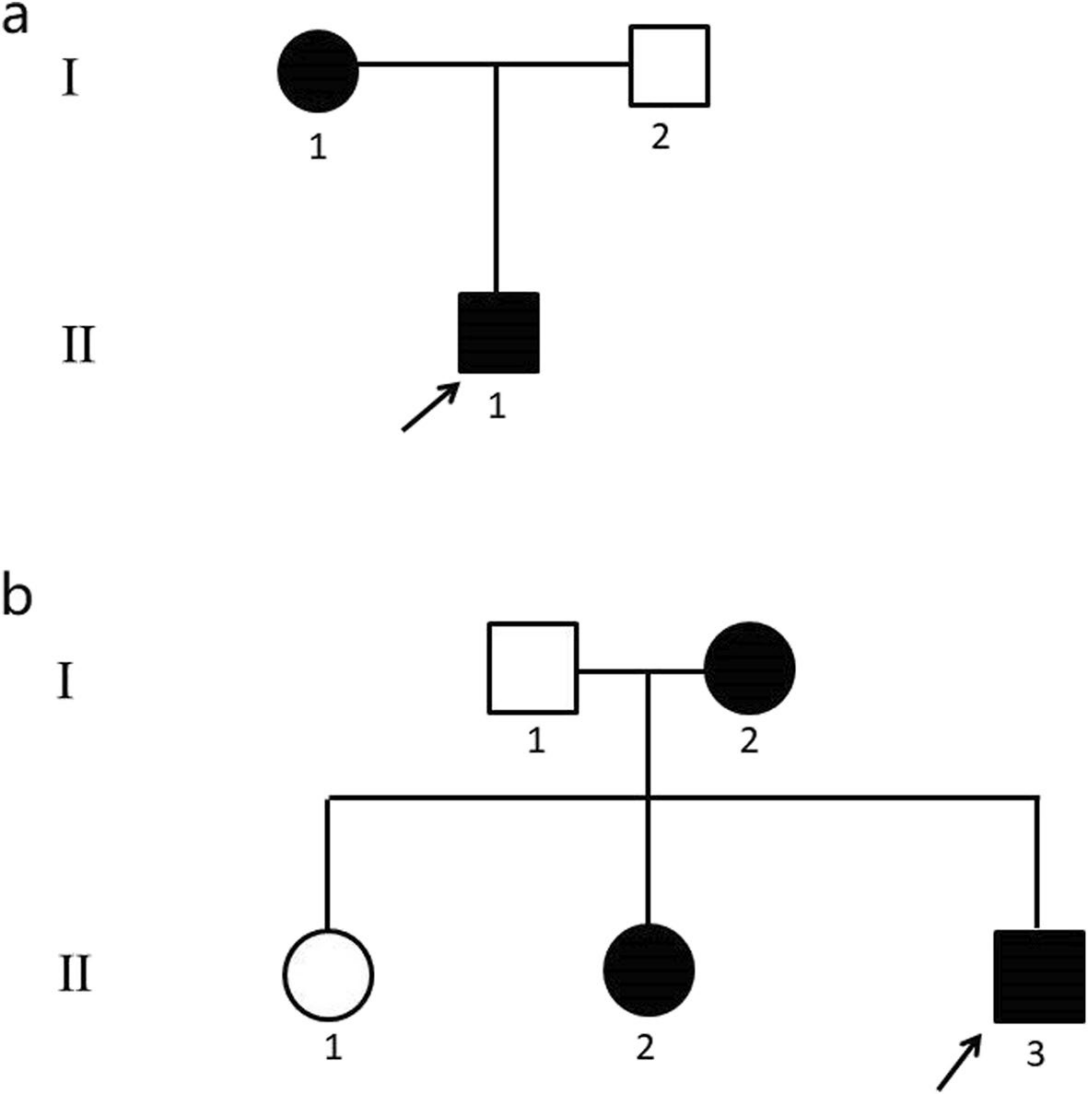
Genogram of the families. In this figure, males are indicated with squares and females are circles. Family members with keratoconus (KC) are represented with solid symbols and normal members are open symbols. **a** Family 1. Subject II.1 in family 1 is the proband (arrow). **b** Family 2. Subject II.3 in family 2 is the proband (arrow)

**Fig. 2:**
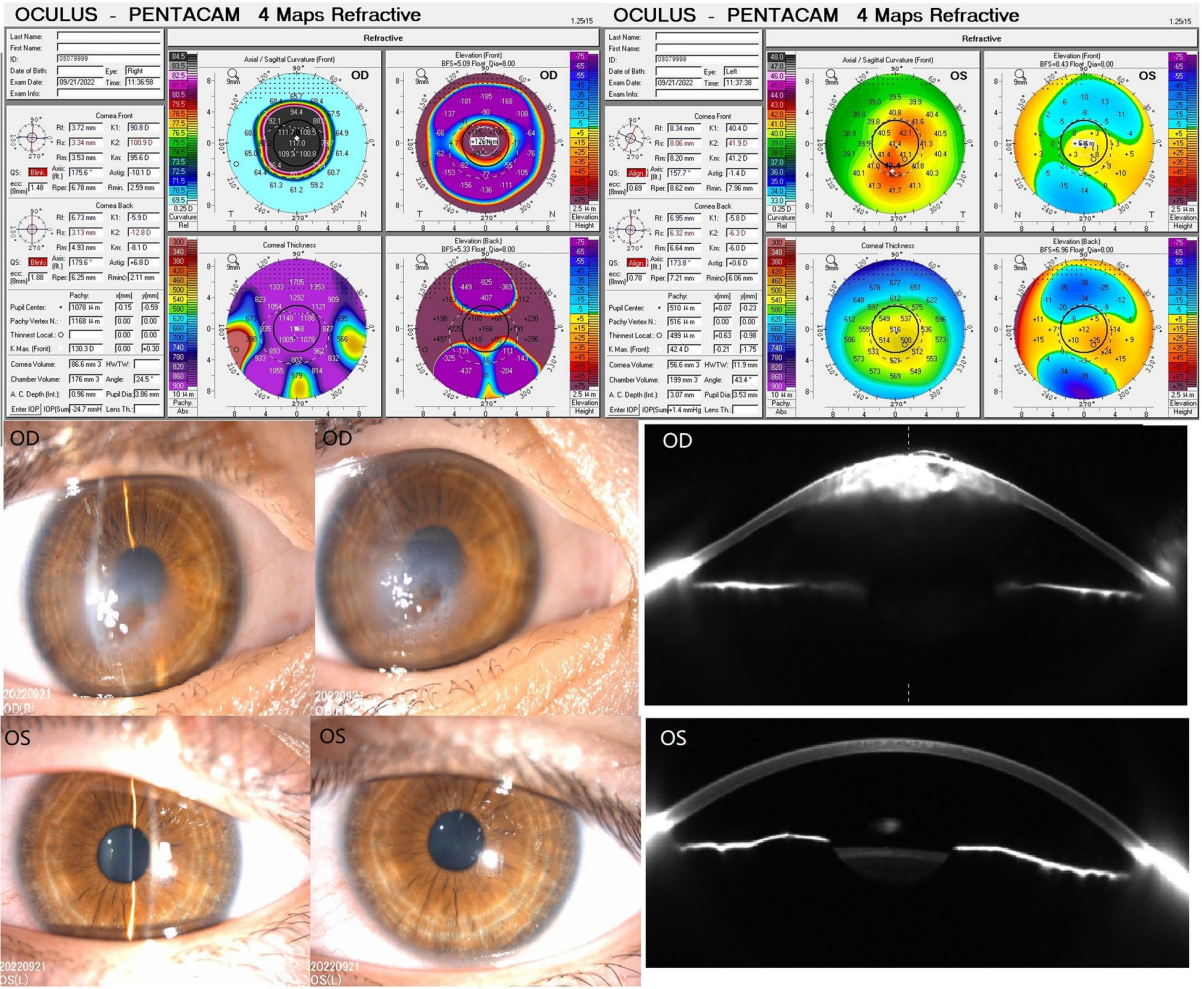
The images of corneal topography, slit lamp examination, and anterior segment optical coherence tomography (AS-OCT) in the proband (subject II. 1) of family 1 are shown. The inferior cornea steepening and irregular astigmatism were observed especially in the right eye (OD). The means of corneal posterior elevation were 166 and 25 µm in the right and left eyes (OS), respectively. Corneal edema and Descemet’s membrane rupture were observed in the right eye

The genograms are presented in Fig. 1 (1a: family 1; 1b: family 2). In family 1, there were two members affected with KC. The proband (II.1) was a 9-year-old boy. He presented with impaired vision, acute keratoconus edema, and rupture of the Descemet’s membrane in the right eye after rubbing the eye 10 days prior. The uncorrected distance vision acuity (UDVA) was 10/50 (right eye) and 20/50 (left eye). His corrected distance vision acuity (CDVA) was 10/50 (right eye) with -3.00 DS/-6.50 DC × 170° and 20/20 (left eye) with -2.00 DS/-1.00 DC × 165° (Fig. 2). The mean posterior elevation of the cornea (PEC) was 166 µm (right eye) and 25 µm (left eye). Another patient from family 1, subject I.1, was a 46-year-old female. Her UDVAs were 10/50 and CDVAs were 20/20 in both eyes, with -3.25 DS/-1.00 DC × 175° and -4.50 DS/-0.50 DC × 10°. The mean PEC was 15 µm in one eye and 16 µm in another. The central corneal thickness (CCT) was 493 and 491 µm. The clinical data of other family members are presented in Figure S1.

**Fig. 3:**
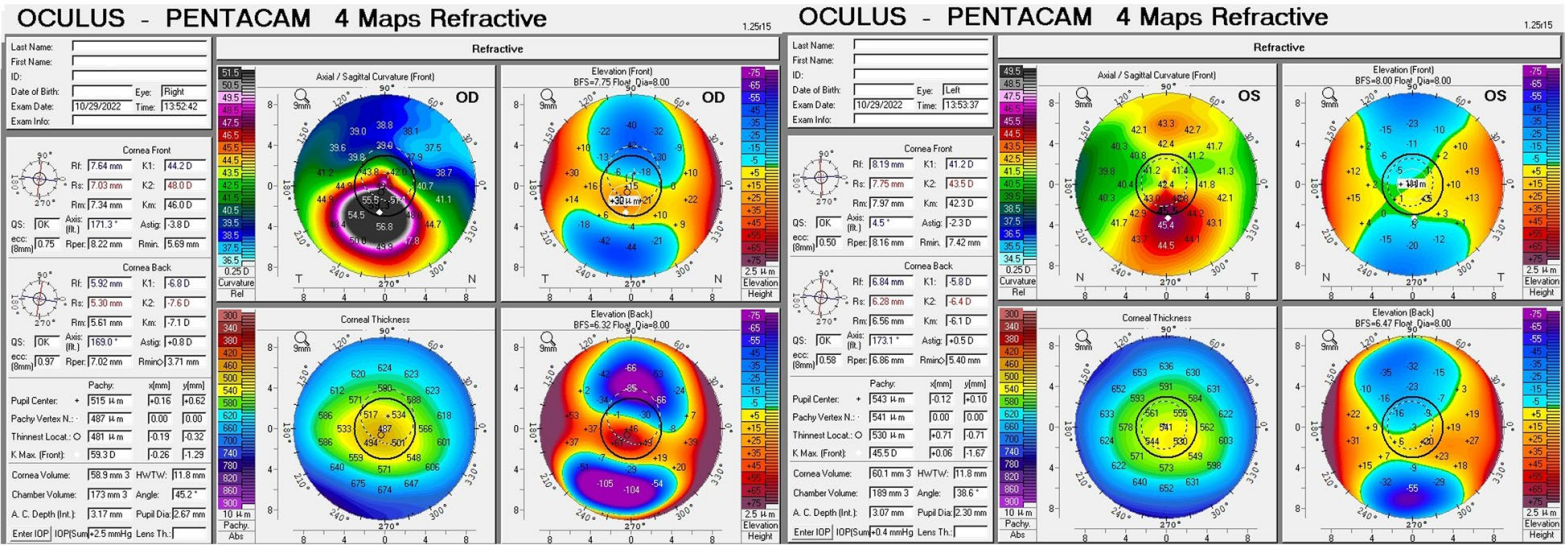
The corneal topography of the proband (subject II. 3) in family 2 is shown. The inferior cornea steepening and irregular astigmatism were observed especially in the right eye (OD). The means of corneal posterior elevation were 61 and 20 µm in the right and left eyes (OS), respectively

In family 2, there were three members afflicted with KC. The proband (II.3) was a 22-year-old male. His UDVAs were 10/50 in both eyes and CDVA was 20/50 (right eye) with -3.50 DS/-4.00 DC × 175° and 20/20 (left eye) with -2.50 DS/-2.00 DC × 5°. The mean PEC and CCT were 61 and 481 µm in the right eye and 20 and 530 µm in the left eye (Fig. 3). Central cornea thinning, inferior steepening, bulging outwards, and irregular astigmatism were observed in both eyes of all three subjects from family 2. The findings of other family members are presented in Figure S2.

The stages of KC subjects in the two families are shown in Table 1. In the 112 sporadic cases, the numbers of patients in different stages were 8 in stage 1, 65 in stage 2, 30 in stage 3, and 9 in stage 4.

**Table 1:** Clinical data of the keratoconus (KC) patients in the two families

KC subjects	Sex	Age at diagnosis	CCT (µm)	Kmax	PEC (µm)	Stages^a^
Family 1						
II.1	M	9	NA (OD), 499 µm (OS)	130.3D (OD), 42.4D (OS)	16 6 µm (OD), 25 µm (OS)	4 (OD) 2 (0S)
I.1	F	46	493 µm (OD), 491 µm (OS)	44.7D (OD), 44.9D (OS)	15 µm (OD), 16 µm (OS)	2 (OD) 2 (0S)
Family 2						
II.3	M	22	481 µm (OD), 530 µm (OS)	59.3D (OD), 45.5D (OS)	61 µm (OD), 20 µm (OS)	3 (OD) 2 (0S)
II.2	F	24	327 µm (OD), 425 µm (OS)	68.6D (OD), 50.8D (OS)	111 µm (OD), 55 µm (OS)	4 (OD) 1 (0S)
I.2	F	50	462 µm (OD), 480 µm (OS)	46.6D (OD), 44.0D (OS)	23 µm (OD), 14 µm (OS)	2 (OD) 2 (0S)

*CCT* Central corneal thickness*, PEC* Posterior elevation of the cornea, *M* Male, *F* Female, *OD* Right eye, *OS* Left eye

^a^Stages: 1) latent stage; 2) incipient stage; 3) completion stage; and 4) scar stage

### Identification and analyses of the novel variant and SNP

A novel variant and an SNP were identified in these two families and not detected in the normal controls.

**Fig. 4:**
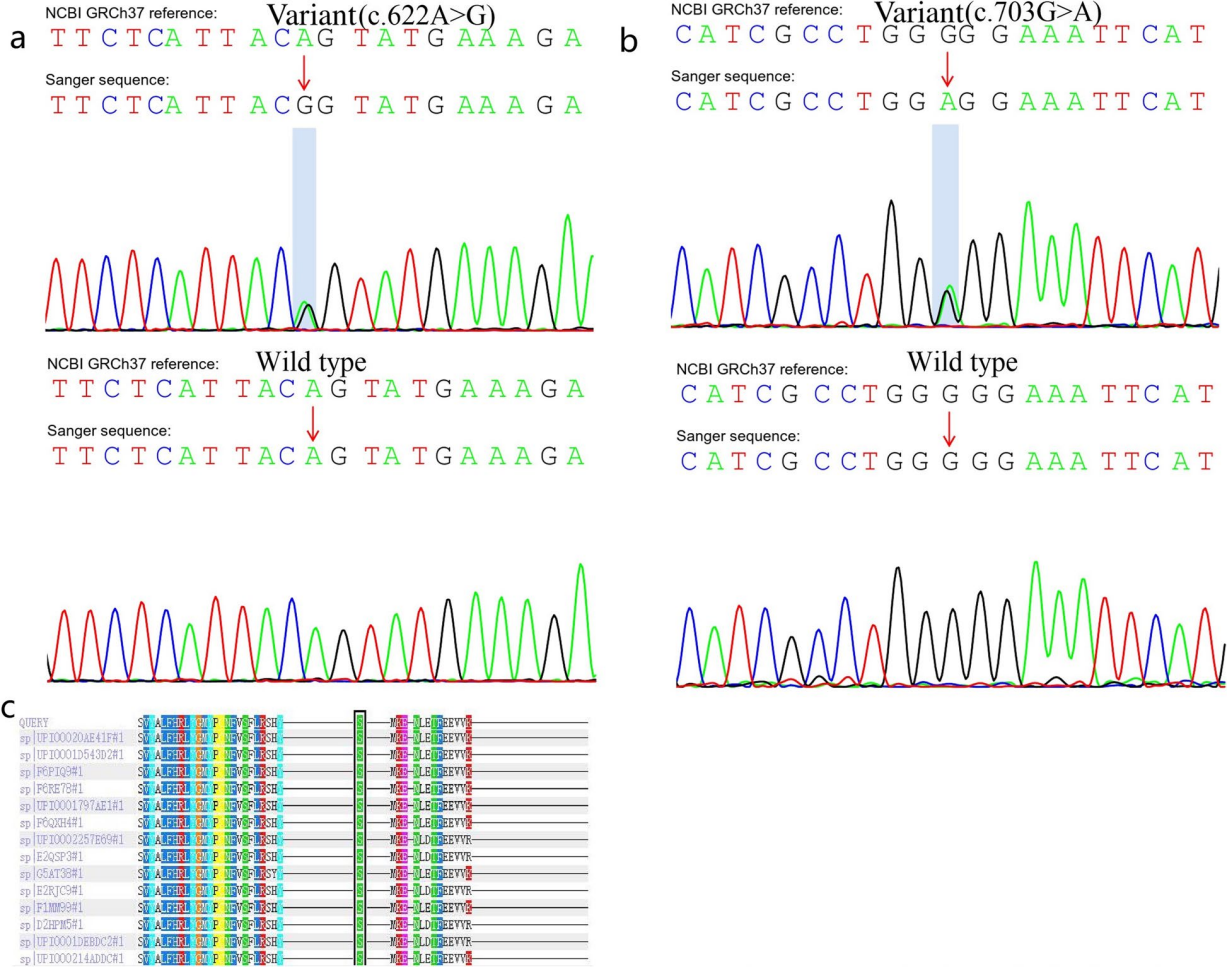
The reports of sequencing and cross-species conservation analysis are shown. **a** The Sanger sequencing in the variant and wild-type samples is shown. The variant c.622A > G in *TSC1* is indicated (arrow). **b** The single nucleotide polymorphism (SNP) rs761232139 in *ALDH3A1* is indicated (arrow). **c** The multiple alignments demonstrate that the arginine at codon 208 in *TSC1* is highly conserved. A black bar highlights the codon 208 in *TSC1*

**Fig. 5:**
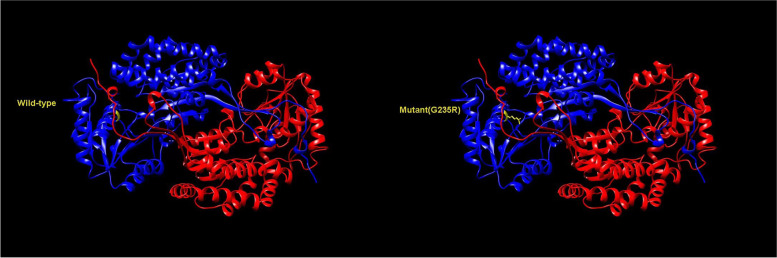
The structures of wild-type *ALDH3A1* and SNP rs761232139 (Gly235Arg) are shown using 3D modeling

In family 1, a variant in *TSC1* was detected, g.135797247A > G (c.622A > G, p.Ser208Gly), which was a missense variant (Fig. 4a). In family 2, an SNP in *ALDH3A1* was detected, rs761232139 (p.Gly235Arg), and was detected in all KC members from family 2 (Fig. 4b). However, there was no significant conformational change of the protein caused by the SNP in *ALDH3A1* compared with the wild-type protein (Fig. 5). The SNP rs761232139 was detected in six patients (5.4%, genotype G/A) from the 112 sporadic KC patients. Compared with the healthy controls, the rs761232139 G/A genotype and A allele frequencies (p = 0.019 and 0.018 respectively, χ2 test,) were higher in the sporadic KC patients.

**Fig. 6:**
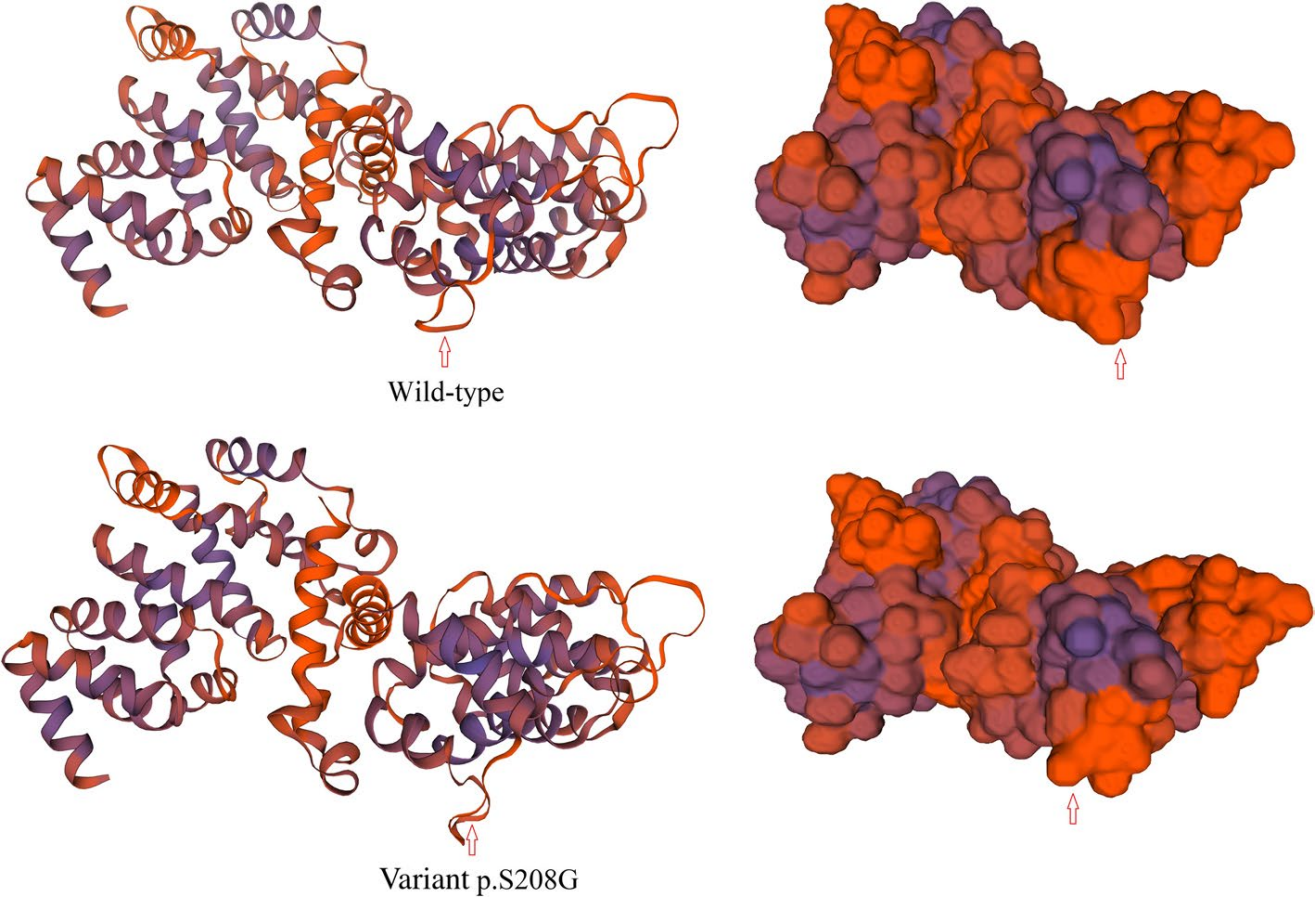
The structures of wild-type *TSC1* and p.Ser208Gly variant are shown using 3D modeling. The differences are indicated by arrows

The variant c.622A > G in *TSC1* was predicted as probably damaging and highly conserved using software programs (Polyphen2, SIFT, PROVEAN, and Mutation Taster) (Table 2 and Fig. 4c). Moreover, the possible conformational alteration of the protein caused by the p.Ser208Gly variant in *TSC1* compared with the wild-type protein was revealed by three-dimensional modeling (Fig. 6). No allele frequency of the variant c.622A > G in *TSC1* was available in the databases of the X1000g2015aug_eas, ExAC_EAS, and gnomAD_exome_EAS. The score of ACMG was uncertain significance and the evidence codes were PS4 and PM2, which means that the variant is rare in the normal population, located in a critical functional domain, and pathogenic supporting via multiple in silico analyses [7]. The allele frequency of the SNP rs761232139 in the normal population was 0.0008 (database: gnomAD_exome_EAS), very rare.

**Table 2:** Identification and analysis of the novel variant

EXON No	Change	Phenotype	Conservation	Polyphen2 prediction	SIFT Prediction	Provean	Mutation Taster Prediction
7	c.622A > G	Nonsynonymous (p.Ser208Gly)	HC	Benign	Deleterious	Deleterious	Disease causing

The p.Ser208Gly *TSC1* variant was screened in the 112 sporadic cases and 100 normal controls. The information is shown in Table 3. Only 1 patient from the 112 sporadic patients had the variant. His clinical data were as follows: 20-year-old male, CCT (415 µm in OD and 462 µm in OS), Kmax (59.6D in OD and 44.1D in OS), PEC (72 µm in OD and 8 µm in OS), CDVA (3/20 in OD and 20/20 in OS), and stage (grade 3 in OD and grade 2 in OS). The SNP rs761232139 in *ALDH3A1* was also screened in the 112 sporadic cases and 100 normal controls. Its distribution of genotype and allele frequencies were significantly different between the sporadic patients and normal controls (*P* < 0.05, Table 4).

**Table 3:** Distribution of the genotype and allele frequencies of c.622A > G in *TSC1* in the 112 sporadic patients and 100 normal controls

Group	Genotype			Allele	
	A/A	A/G	GG	A	G
Patients	111 (99.1%)	1 (0.9%)	0 (0%)	223 (99.6%)	1 (0.4%)
Controls	100 (100%)	0 (0%)	0 (0%)	200 (100%)	0 (0%)

Patients vs. Controls: χ2 test, *P* = 0.35

**Table 4:** Distribution of the genotype and allele frequencies of rs761232139 in *ALDH3A1* in the 112 sporadic patients and 100 normal controls

Group	Genotype			Allele	
	G/G	G/A	AA	G	A
Patients	106 (94.6%)	6 (5.4%)	0 (0%)	218 (97.3%)	6 (2.7%)
Controls	100 (100%)	0 (0%)	0 (0%)	200 (100%)	0 (0%)

Patients vs. Controls: χ^2^ test, *P* = 0.02

## Discussion

KC is a complex disorder caused by multifactorial etiology, such as the interaction among behavioral, environmental, and genetic factors. Several genes associated with the familial form of KC have been studied and few mutations have been reported. Some of the familial forms of KC are related to other genetic disorders such as Down syndrome, connective tissue disorders, and Leber congenital amaurosis [1–6, 8]. Investigating the various KC subsets could shed light on its etiology. In this study, two findings that may be associated with a familial KC were identified: a missense variant in *TSC1* and an SNP in *ALDH3A1.*

*TSC1* and *TSC2* genes were named because some of their mutations are related to a genetic disorder TSC [9–12]. TSC is a disorder that causes hamartomas in multiple organs (e.g., brain, eyes, skin, kidneys). The pathogenesis of TSC involves changes in cellular proliferation and differentiation. Though rare, the co-existence of TSC and KC has been reported [9–11] and a 4-base-pair deletion in *TSC2* was identified [11]. In the present investigation, the variant of g.135797247A > G (c.622A > G, p.Ser208Gly) located in exon 7 of *TSC1* gene was detected. This variant may cause a conformational change in the protein and has not been previously reported in patients with or without TSC. The residue that this variant involves has been considered a distinct functional and/or structural sequence of the *TSC1* gene. Moreover, the variant was predicted as probably damaging and highly conserved among species. In the family in which the novel variant was detected, TSC symptoms were not found in any family member.

TSC1 protein (hamartin) interacts with other proteins including TSC2 protein (tuberin), TBC17D, AKT2, MTOR, and RPS6KA1 to exert its effects [9–13]. The complex of TSC1, TSC2, and TBC17D proteins negatively regulates cellular proliferation and differentiation. The underlying mechanism is a canonical signaling pathway with Ras homologue enriched in the brain (Rheb) and the mammalian target of rapamycin complex 1 (mTORC1) involved [9–13]. AKT2, MTOR, and RPS6KA1 were screened as potential interactors of TSC1 protein using software [13] and were found to be expressed in ocular tissues [9]. All of these findings indicate that TSC1 protein plays a role in the eye. The alterations in the structure and function of TSC1 protein may change its interaction with other proteins and affect cell growth and proliferation in the eye. This may cause KC, a disorder of corneal stroma hypoplasia [8], in patients with or without TSC.

*ALDH3A1* is thought to be involved in KC etiology [14–16]. ALDH3A1 SNPs, rs1042183 and rs2228100, are strongly associated with KC in the Polish and Korean populations [14, 16]. The association of ALDH3A1 with intraocular pressure [17] and refractive astigmatism [18, 19] have also been observed. Furthermore, the relationship between astigmatism and KC has been reported, as 14.1% of patients with more than 2D astigmatism suffer from KC [20]. Our findings further demonstrated the association of ALDH3A1 with KC. A SNP in *ALDH3A1* was detected, rs761232139 (p.Gly235Arg). This SNP is located in exon 6 of *ALDH3A1* gene and is predicted as highly conserved among species and probably damaging. The SNP was identified in all KC members from the family 2, and in six sporadic KC patients (5.4%) from the total 112 patients but none of the normal controls, which means the detection rate of this SNP (rs761232139) in KC patients is much higher than that in normal people. Thus, SNP (rs761232139) identified in this study was considered to increase the risk of KC occurrence and inherited in a dominant model. The residue that the SNP involves was considered a distinct structural and/or functional sequence of ALDH3A1, which is adjacent to the active site Cys-243.

ALDH3A1 protein is a very important enzyme that is highly expressed in the corneal epithelium [15, 21]. It protects the cornea from ultraviolet (UV)-induced damage in various aspects such as absorbing UV radiation directly, metabolizing toxic aldehydes produced by UV-induced lipid peroxidation, and eliminating UV-produced reactive oxygen species (ROS) [22]. The association of accumulated ROS and toxic aldehydes with KC has been reported in previous studies [22–26]. Furthermore, the SNP identified in the present study is quite close to the active site Cys-243 in ALDH3A1. This cysteine residue plays an important role in the aldehydic carbonyl group attack, formation of a covalent thiohemiacetal intermediate and NAD(P) + , and interaction with Glu-209 and Glu-333 in ALDH3A1 [27]. These functions are involved in the elimination of ROS accumulation and subsequent damage to the cells, which protects the cornea from UV radiation injury [22, 28].

## Conclusions

In conclusion, a novel variant c.622A > G in *TSC1* and a SNP rs761232139 in *ALDH3A1* have been detected in two Chinese KC families. These two findings may play a role in the pathogenesis of KC. These findings expand the spectrum of identified genetic characteristics in *KC*. Further studies with a larger sample size are warranted to confirm the findings and reveal the mechanism by which they cause KC.

### Supplementary Information

**Additional file 1: Figure S1.** a: The corneal topography (Pentacam) reports for I.1 in family 1. b: the Corvis ST report for I.1 in family. **Figure S2.** a: The corneal topography (Pentacam) reports for I.2 in family 2. b: The corneal topography (Pentacam) reports for II.2 in family 2.

## Data Availability

The datasets generated and analyzed during the current study are available in the NCBI ClinVar repository, ClinVar accession number: SCV003804500 and accessible direct link: https://www.ncbi.nlm.nih.gov/clinvar/variation/2442791/?oq=SCV003804500&m=NM_000691.5(ALDH3A1):c.703G%3EA%20(p.Gly235Arg). ClinVar accession number: SCV003804463 and accessible direct link: https://www.ncbi.nlm.nih.gov/clinvar/variation/2430291/?oq=SCV003804463&m=NM_000368.5(TSC1):c.622A%3EG%20(p.Ser208Gly).

## Abbreviations

KC: Keratoconus;
*TSC1*: Tuberous sclerosis 1
*ALDH3A1*: Aldehyde dehydrogenase 3 family member A1
ES: Exome sequencing
PCR: Polymerase chain reaction
