# Supplementary material for: Identification of genetic variants in two families with Keratoconus
Source: BMC Med Genomics. 2023 Nov 21;16:299. doi: 10.1186/s12920-023-01738-x (PMC10664684; doi:10.1186/s12920-023-01738-x)
Supplement: Supplementary file 1 — Additional file 1: Figure S1. a: The corneal topography (Pentacam) reports for I.1 in family 1. b: the Corvis ST report for I.1 in family. Figure S2. a: The corneal topography (Pentacam) reports for I.2 in family 2. b: The corneal topography (Pentacam) reports for II.2 in family 2. [file 12920_2023_1738_MOESM1_ESM.doc]

**
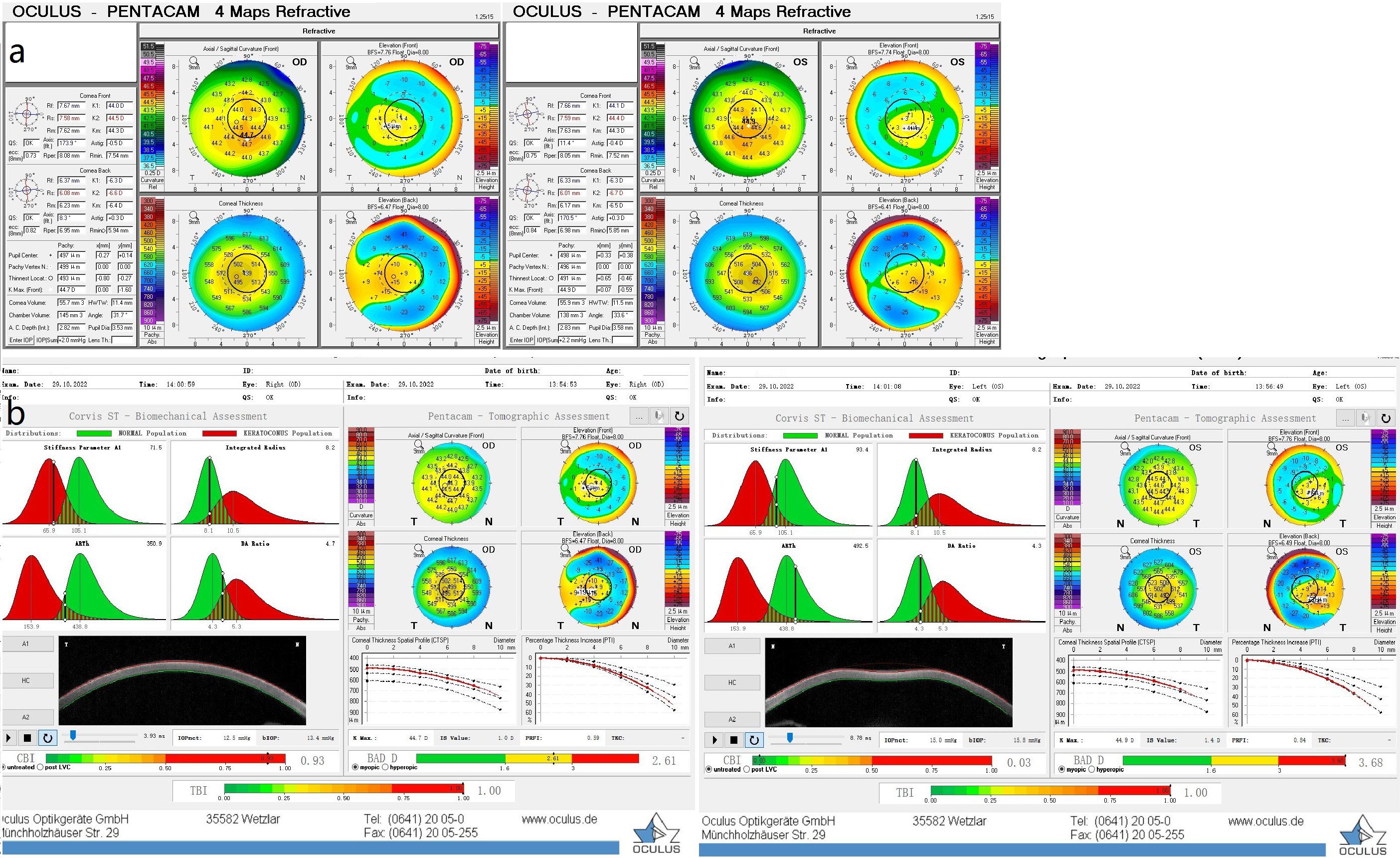
**

**Figure S1.** a: The corneal topography (Pentacam) reports for I.1 in family 1

b: the Corvis ST report for I.1 in family


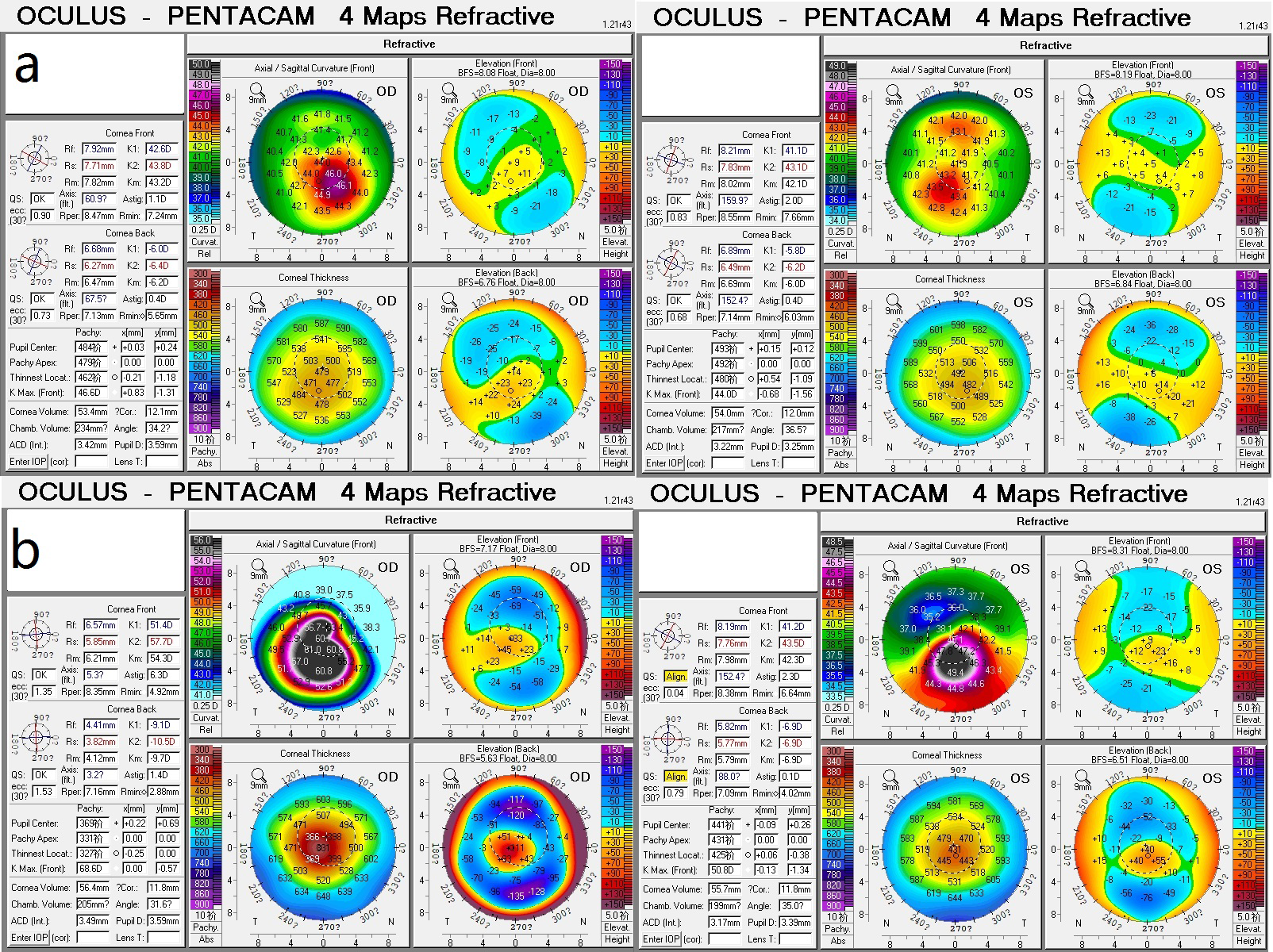


**Figure S2.** a: The corneal topography (Pentacam) reports for I.2 in family 2

b: The corneal topography (Pentacam) reports for II.2 in family 2
